# Ceramide in the Molecular Mechanisms of Neuronal Cell Death. The Role of Sphingosine-1-Phosphate

**DOI:** 10.1007/s12035-013-8606-4

**Published:** 2014-01-14

**Authors:** Kinga Czubowicz, Robert Strosznajder

**Affiliations:** Laboratory of Preclinical Research and Environmental Agents, Department of Neurosurgery, Mossakowski Medical Research Centre, Polish Academy of Sciences, 5 Pawinskiego Street, 02-106 Warsaw, Poland

**Keywords:** Ceramide, S1P, PARP-1, AIF, SH-SY5Y

## Abstract

Ceramide and sphingosine-1-phosphate (S1P), two important bioactive sphingolipids, have been suggested as being key players in the pathology of Alzheimer’s disease in inflammation and cancer. However, their role in the molecular mechanisms of neuronal death has not been fully elucidated. Our study indicated that ceramide significantly enhanced the level of free radicals and decreased the viability of the human *neuroblastoma* cell line (SH-SY5Y) through inhibition of the prosurvival PI3-K/Akt pathway. Ceramide also decreased anti-apoptotic (Bcl-2) and increased pro-apoptotic (Bax, Hrk) mRNA/protein levels. Concomitantly, our study indicated that ceramide induced poly(ADP-ribose) polymerase-1 (PARP-1) activation and accumulation of poly(ADP-ribose) PAR, a signalling molecule involved in mitochondria-nucleus cross-talk and mitochondria integrity. Ceramide treatment significantly decreased the level of apoptosis-inducing factor (AIF) in the mitochondria. The PARP-1 inhibitor (PJ-34) prevented AIF release from the mitochondria. In addition, our data showed that exogenously added S1P increased the viability of SH-SY5Y through the S1P (1,3) receptor-dependent mechanism. It was also revealed that the S1P and PARP-1 inhibitor (PJ-34) decreased oxidative stress, gene expression of the pro-apoptotic Hrk protein and up-regulated the anti-apoptotic Bcl-2 protein. Our data demonstrate that neuronal cell death evoked by ceramide is regulated by PARP/PAR/AIF and by S1P receptor signalling. In summary, our results suggest that PARP-1 inhibitor(s) and modulators of sphingosine-1-phosphate receptor(s) should be considered in potential therapeutic strategies directed at neurodegenerative diseases.

## Introduction

Ceramides are a class of sphingolipids that are abundant in cell membranes and play an important role in regulation of the fluidity and structure of the lipid bilayer. During the last decade, ceramides have been recognised as very important second messengers for multiple extracellular stimuli mediating many cellular processes, mainly those associated with the regulation of cell proliferation, differentiation, growth arrest and apoptosis [1–3]. Ceramides are composed of a sphingoid base linked to a fatty acid of varying chain length (14–26 carbons) via an amide bond. Cell-permeable short chain ceramide, C2-ceramide and C6-ceramide have been used in many studies to mimic ceramide-mediated cell death pathways. It was shown that C2-ceramides were present naturally in the brain at low levels [4]. Treatment of neuronal cells with ceramides at low concentration promotes cell differentiation, survival and neurite outgrowth, while at higher concentration it induces apoptosis. Downstream targets of ceramides are diverse and not completely characterised. It has been described that ceramide regulates mitogen-activated protein kinase (MAPK), protein kinase ζ (PKCζ), stress-activated protein kinases (SAPK/c-Jun N-terminal kinases (JNK)), ceramide-activated protein kinases (CAPK), ceramide-activated protein phosphatase (CAPP) and phospholipases (cPLA, PLD, PLA_2_) [5–9]. Ceramides are generated by three metabolic pathways: *de novo* synthesis, sphingomyelin hydrolysis and the salvage pathway [1]. These pathways are activated in response to oxidative stress, tumour necrosis factor-α, chemotherapeutic agents and radiation. Ceramide may be broken down by ceramidases, thus leading to the formation of sphingosine, which can be phosphorylated by sphingosine kinases (Sphk1and Sphk2) to sphingosine-1-phosphate (S1P). It is known that S1P acts in an autocrine/paracrine manner via a family of five S1P-specific cell-surface G-protein-coupled receptors (GPCRs, termed S1P1-5) [10, 11]. S1P signalling through these receptors activates the PI3K/Akt pathway and Ras/extracellular signal-regulated kinases (ERK) to promote proliferation and to prevent apoptosis [12, 13]. PI3K/Akt regulates many cellular processes including metabolism, proliferation, cell survival, growth and angiogenesis [14]. The balance between the levels of ceramide and S1P, called “ceramide/S1P rheostat”, contributes to the fate of the cells [15]. Ceramides, through the formation of channels, have been reported to induct mitochondrial outer membrane permeabilisation, which is a key event in apoptotic signalling [16, 17]. Ceramides affect the activities of the mitochondrial electron transport chain and lead to cellular energy crisis [18, 19]. This mitochondrial failure evoked by ceramide leads to enhancement of oxidative stress, DNA damage and activation of nuclear enzyme poly(ADP-ribose) polymerase-1 (PARP-1). It was reported previously that the formation of long-chain poly(ADP-ribose) (PAR) and the release of the apoptosis-inducing factor (AIF) from mitochondria to nucleus may accelerate cell death [20–25].

A number of studies have indicated the role of abnormal sphingolipid metabolism in brain ischemia, inflammation, Alzheimer’s disease (AD) and other neurodegenerative disorders [26–32]. It was observed in in vivo studies that both a decrease in the S1P level and ceramide accumulation were proportional to the degree of cognitive impairment, loss of intellectual ability and loss of neurons [27, 33, 34]. Ceramides have been indicated as key player in neuronal cell death; however, their role is not yet well understood. It has been demonstrated that neuronal death induced by ceramide may be linked to the caspase-9/caspase-3 regulated intrinsic apoptotic pathway. The data indicated that C2-ceramide in rat primary cortical neurons induces up-regulation of active caspase-9 and caspase-3 protein levels [35, 36]. On the other hand, Kim et al. [37], in human neuroblastoma cell line SY-SY5Y, observed that the inhibition of caspases was not sufficient to attenuated ceramide-induced cell death. Ceramide has been also involved in the control of autophagy [38]. It has been observed that ceramides activate protein phosphatase PP2A, which in turn blocks Akt (activation) the well-known autophagy suppressor [39]. However, the molecular mechanisms of death signalling pathway evoked by ceramide are not fully elucidated. Additionally, the role of exogenous S1P in neuroprotection is still under investigation.

In this study, we investigated the molecular mechanism of neuronal cell death evoked by ceramide focusing on the PI3K/Akt/GSK-3β pathway and on PARP-1/AIF signalling in the human *neuroblastoma* cell line (SH-SY5Y). The response of pro-apoptotic and anti-apoptotic gene expression was analysed. Moreover, the neuroprotective effect of S1P was evaluated.

## Experimental Procedures

### Cell Culture

The studies were carried out using the human *neuroblastoma* cell line (SH-SY5Y) (a kind gift from Prof. Anne Eckert, Neurobiology Laboratory for Brain Aging and Mental Health, Psychiatric University Clinics, University of Basel). The SH-SY5Y cells were used for experiments between 5 and 15 passage numbers, cultured in MEM/F-12 Ham Nutrient Mixtures (1:1) supplemented with 15 % heat-inactivated fetal bovine serum (FBS), 1 % penicillin/streptomycin and 2 mM glutamine. Cells were maintained at 37 °C in a humidified incubator containing 5 % CO_2_. For the experiment, confluent cells were sub-cultured into dishes or collagen coated 96-well plates. Prior to treatment, the cells were cultivated in low serum (2 % FBS) medium and then were treated with different inhibitors and compounds.

### Cell Treatment Protocols

SH-SY5Y cells were treated with cell-permeable, biologically active ceramide, C2-ceramide, at a 10 to 50 μM concentration for various times up to 24 h. In most of the experiments, the cells were treated with the following compounds: PJ-34 (20 μM), PARP-1 inhibitor; α-pifithrin (20 μM), p53 inhibitor; cyclosporine A (2 μM), inhibitor of the permeability transition pore; UO126 (1 μM), ERK1/2 kinase inhibitor; SP600125 (5 μM), JNK kinase inhibitor; sphingosine-1-phosphate (1 μM), LY294002 (50 μM), PI3-K inhibitor and Z-DEVD-FMK (100 μM), caspase-3 inhibitor added 1 h before incubation with C2-ceramide (25 μM). Our data showed that most of these compounds had no effect on cell viability in the control condition, with the exception of PARP-1 inhibitor, JNK kinase inhibitor and PI3-K inhibitor, which significantly decreased SH-SY5Y cell survival (by about 20 %, PJ-34, SP600125; 35 %, LY294002). The above-mentioned compounds were purchased from the following: C2-ceramide and sphingosine-1-phosphate from Enzo Life Sciences, LY294002 from Cell Signalling Technology, PJ-34, α-pifithrin, SP600125, U0126 and cyclosporine A from Sigma-Aldrich, Z-DEVD-FMK from Tocris Bioscence.

### Cell Viability Analysis

Cell viability and mitochondrial function were evaluated using 2-(4,5-dimethylthiazol-2-yl)-2,5-diphenyltetrazolium bromide (MTT). After 24 h incubation with C2-ceramide and selected compounds, MTT was added to all of the wells. The cells were incubated at 37 °C for 2 h, followed by cell lysis and spectrophotometric measurement at 595 nm.

### Isolation of Cytosolic, Mitochondrial and Nuclear Fractions

Cells were washed and scraped into ice-cold phosphate buffered saline (PBS) and were pelleted at 1,000×*g* for 3 min at 4 °C. The pellet was resuspended in hypotonic buffer (10 mM Tris–HCl, pH 7.4, 1 mM EDTA, 1 mM EGTA, 1 mM dithiotreitol, 1.5 mM MgCl_2_, 10 mM KCl and protease inhibitors); cell membranes were disrupted by homogenisation and were pelleted at 500×*g* for 10 min at 4 °C. The pellet (P1, the crude nuclear fraction) was resuspended in 25 mM Tris–HCl pH 7.4 with protease inhibitors and was used for Western blot analysis. A supernatant (S1) was used for isolation of the mitochondria and the cytosolic fraction by centrifugation at 15,000×*g* for 10 min at 4 °C. The pellet (P2) (crude mitochondria) was resuspended in 25 mM Tris–HCl pH 7.4 with protease inhibitors and was used for Western blot analysis.

### Measurement of PARP-1 Activity

PARP-1 activity was determined using ^14^C-labelled βNAD^+^ as a substrate. The incubation mixture, in a final volume of 100 μl, contained 200 μM (adenine-^14^C)βNAD^+^ (4 × 10^5^ dpm, Amersham Biosciences), 100 mM Tris–HCl buffer (pH 8.0), 10 mM MgCl_2_, 5 mM DTT, 50 μM p-APMSF and 50–100 μg of protein. The mixture was incubated for 1 min at 37 °C and the reaction was stopped with 0.8 ml of ice-cold 25 % trichloroacetic acid (TCA). Precipitates were collected on Whatman GF/B filters, washed three times with 5 % TCA and left overnight for drying. The radioactivity was measured using an LKB Wallach 1409 scintillation counter.

### Determination of Free Radicals

The fluorescent 2′,7′-dichlorofluorescein (DCF) fluorescence assay detects the level of hydrogen peroxide and other reactive oxygen species (ROS) in cells. Free radicals were determined based on ROS-mediated conversion of 2′,7′-dichlorodihydrofluorescein diacetate (H2DCFDA) into DCF [40, 41]. SH-SY5Y cells were treated with C2-ceramide and selected inhibitors for 24 h and then loaded with 10 μM H2DCFDA in dimethyl sulfoxide (DMSO) by being incubated for 50 min at 37 °C in Hank’s buffer without Phenol Red (Sigma–Aldrich, St. Louis MO, USA). DMSO was used at a final concentration of 0.05 %; at this concentration it had no effect on the free radical levels. Fluorescence of DCF was measured using a Perkin Elmer LS 50B spectrofluorometer with excitation and emission wavelengths at 488 and 535 nm, respectively.

### Lactate Dehydrogenase Assay Kit

For the analysis of necrotic cell death, a lactate dehydrogenase (LDH) assay was performed using a commercial LDH cytotoxicity Assay Kit II (BioVision) according to manufacturer’s instructions. After 24 h incubation with C2-ceramide, PJ-34 and S1P cells were centrifuged at 600 g for 10 min. Next, the clear medium solution was transferred into 96-well plate. To each well, 100 μl LDH reaction mix were added. After 30 min incubation at room temperature, the absorbance at 450 nm was measured.

### Immunochemical Determination of the Protein Level

After protein measurement according to Lowry, the homogenate of SH-SY5Y or the mitochondrial fraction was mixed with 5× Laemmli sample buffer and denatured for 5 min at 95 °C. Forty to 60 μg of the protein was loaded per lane on 10 % acrylamide gels and examined by sodium dodecyl sulfate (SDS)–polyacrylamide gel electrophoresis. The proteins were transferred onto polyvinylidene difluoride membranes at 100 V. The membranes were incubated in 5 % dry milk in TBS with Tween 20 (TBS-T) for 1 h and exposed overnight to the following antibodies: anti-AIF (from Santa Cruz Biotechnology, CA, USA), anti-Bcl-2 (from Sigma-Aldrich, St. Louis, MO, USA), anti-pBad, anti-Bad (from Santa Cruz Biotechnology), anti-GAPDH (from Sigma–Aldrich), and anti-Actin (from MP Biomedicals. CA, USA). After treatment for 1 h with corresponding horseradish peroxidase-coupled secondary antibodies (anti-rabbit from Sigma–Aldrich or anti-mouse from Amersham Biosciences), the protein bands were detected by ECL reagent (ThermoScientific). After detection, the membranes were treated with stripping buffer (50 mM glycine, pH 2.5, 1 % SDS) for further blots.

### Analysis of PAR Immunoreactivity

For the immunochemical detection of PAR formed in vitro, the cells where homogenized in a cold buffer containing 10 mM Tris–HCl pH 8.0, 50 mM NaCl, 1 mM EDTA, 1 mM DTT and protease inhibitors. The protein homogenate was pre-incubated for 5 min at 37 °C and then incubated for 1 min in the presence of 100 mM Tris–HCl pH 8.0, 10 mM MgCl_2_, 5 mM DTT and 0.2 mM βNAD^+^ (a blank sample was incubated without βNAD^+^). The reaction was terminated with denaturing sample buffer (5 min at 95 °C) and the material was subjected to electrophoresis and then transfer as described above. The membrane was blocked in 5 % dry milk and probed with 1:400 anti-PAR antibody (Alexis Corp., clone#10H) and then with 1:2,000 secondary horseradish peroxidase-linked anti-rabbit IgG. The bands were visualised using the ECL kit and the level of PAR was measured densitometrically.

### Analysis of the mRNA Level

RNA was isolated using TRI-reagent from Sigma-Aldrich. The isolated RNA was dissolved in RNAse-free water (Applied Biosystems, Foster City, CA, USA). The amount and purity of RNA was determined using spectrophotometric measurement at 260 and 280 nm. The OD260/OD280 ratio 256 of the RNA samples ranged from 1.6 to 1.9. Isolated RNA (5 μg) was used in reverse transcription polymerase chain reaction. Reverse transcription was performed by using a High-Capacity cDNA Reverse Transcription Kit according to the manufacturer’s protocol (Applied Biosystems). Quantitative PCR was performed on an ABI PRISM 7500 apparatus by using pre-developed TaqMan Gene Expression Assays (Applied Biosystems)*: actb* Hs99999903_m1; *bax* Hs00180269_m1; *bcl-2* Hs00608023_m1; *hrk* Hs02621354_s1 according to the manufacturer’s instructions. Actb was selected and used in all of the studies as a reference gene. The relative level of mRNA was calculated by the ΔΔCt method.

### Hoechst Immunostaining

For morphological studies, SH-SY5Y cells were subjected for 24 h to oxidative stress evoked by C2-ceramide together with cytoprotective compounds. Coverslips containing SH-SY5Y cells were collected and washed in PBS. Nuclei were visualised with Hoechst 33342 291 (0.2 μg/ml, Riedel-de-Haën Germany) fluorescent staining. The cells were examined under a fluorescence microscope (Olympus BX51, Japan) and photographed with a digital camera (Olympus DP70, Japan).

### Statistical Analysis

Statistical analyses between two groups were conducted using Student’s *t* test. Analyses among multigroup data were conducted using one-way analysis of variance (ANOVA), followed by the Newman–Keuls post hoc test. The data are given as the means ± SEM. *p* values < 0.05 were considered statistically significant.

## Results

**Fig. 1:**
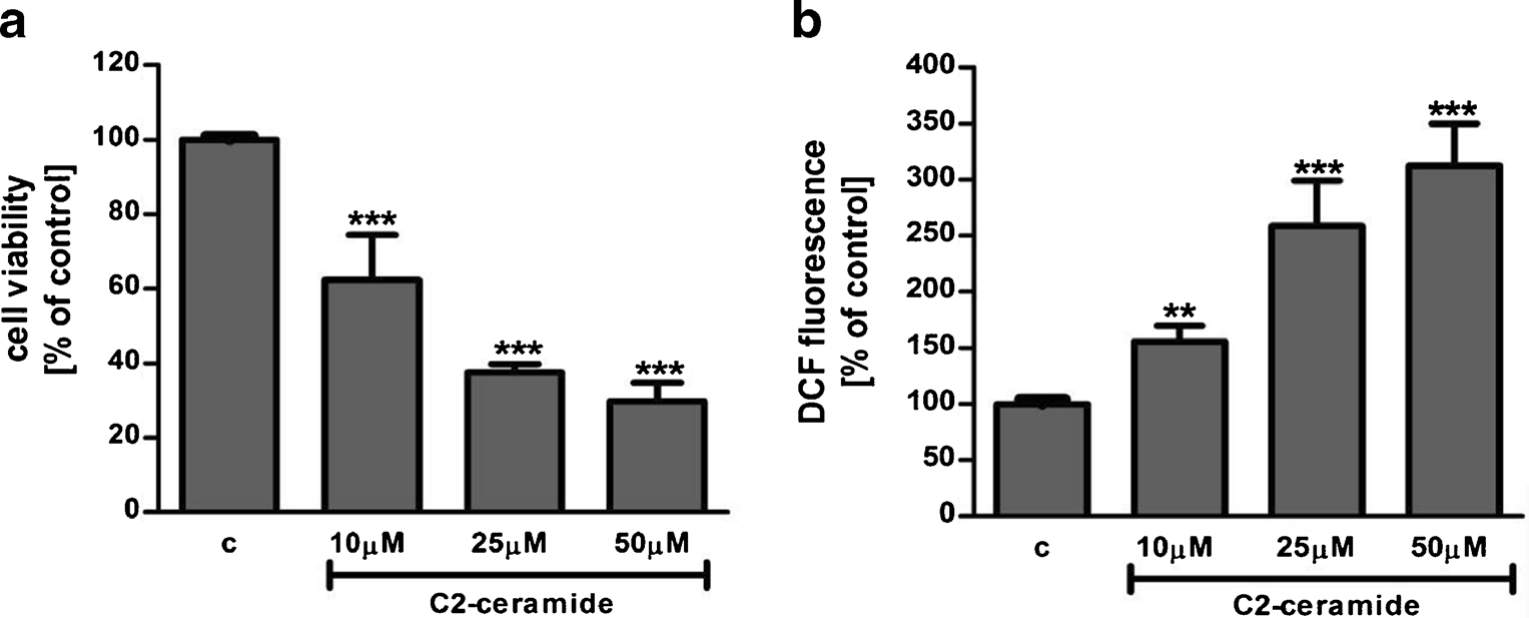
The effect of C2-ceramide on SH-SY5Y cell viability (**a**) and ROS generation (**b**) after 24 h incubation. Data represent the mean value ± SEM for three separate experiments with four to six replications (**a**) and for three separate experiments with three replications (**b**). ^***^ *p* < 0.001, ^**^ *p* < 0.01 versus control SH-SY5Y cells by using one-way ANOVA followed by the Newman-Keuls post hoc test

**Fig. 2:**
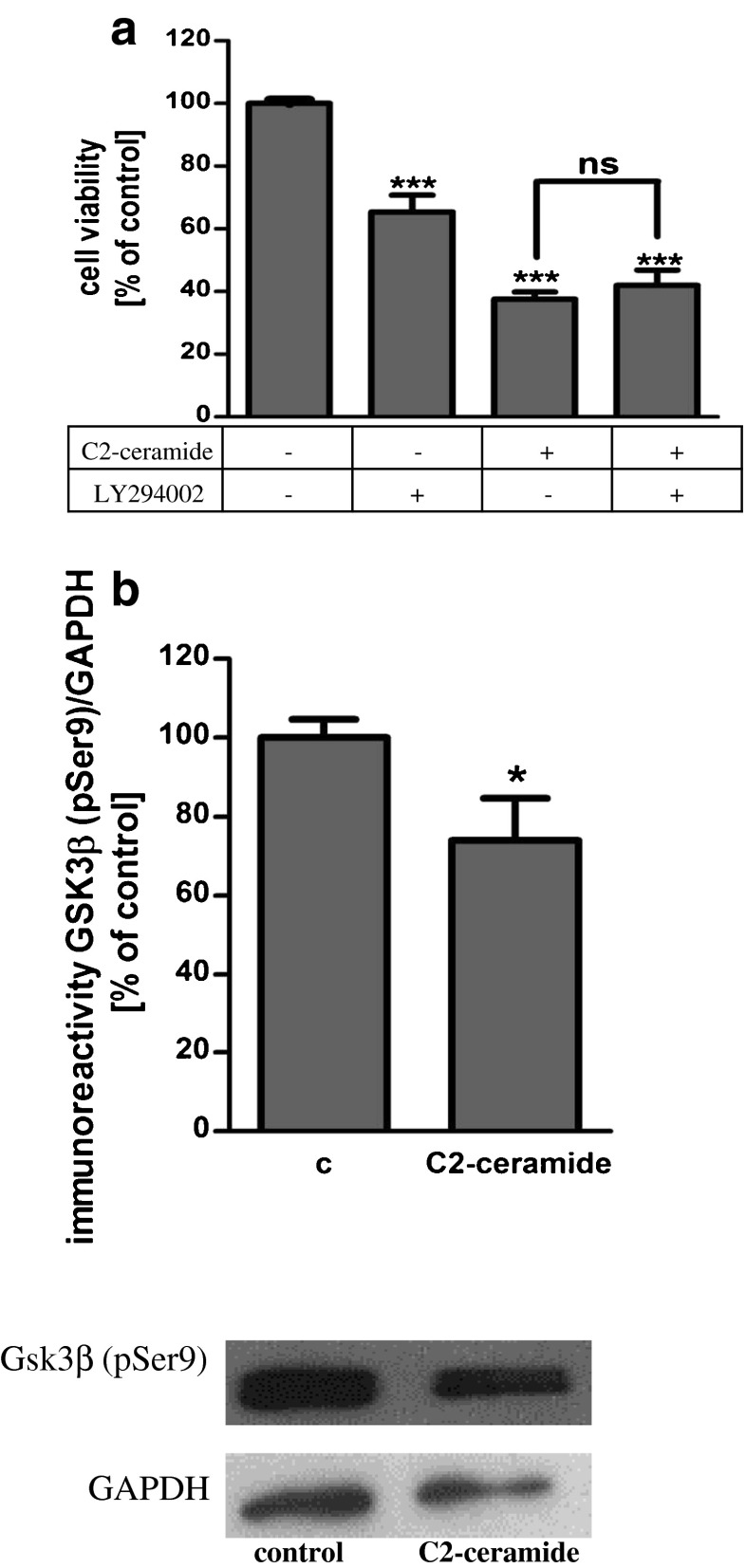
The effect of PI3-K kinase inhibitor (LY294002 {50 μM}) on SH-SY5Y cell viability under oxidative stress evoked by C2-ceramide (25 μM, 24 h) (**a**). Data represent the mean value ± SEM for three separate experiments with four to six replications. ***p < 0.001 versus control SH-SY5Y cells by using one-way ANOVA followed by the Newman-Keuls post hoc test. The effect of C2-ceramide (25 μM, 24 h) on the level of GSK3β (pSer 9) immunoreactivity (**b**). Data represent the mean value ± SEM for three separate experiments normalised against GAPDH. Representative Western blots from one typical experiment are shown below the graphs. *p < 0.0452 versus control SH-SY5Y cells by Student’s *t* test

**Fig. 3:**
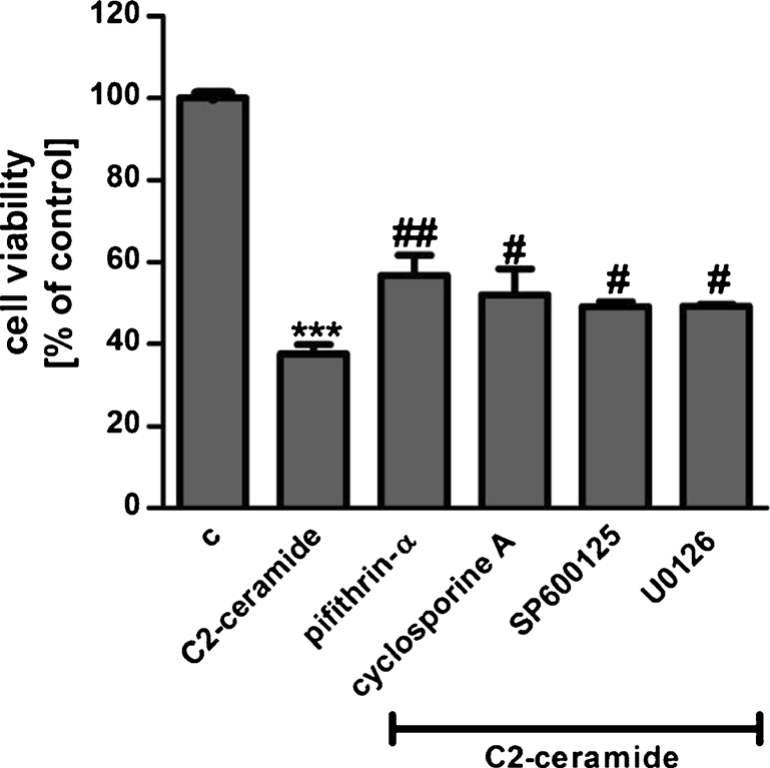
Evaluation of selected molecular events in neuronal cell (SH-SY5Y) death evoked by C2-ceramide (25 μM, 24 h). The following specific inhibitors were used: α-pifithrin (20 μM), p53 inhibitor; cyclosporine A (2 μM), inhibitor of the mitochondrial permeability transition pore; UO126 (1 μM), inhibitor of ERK1/2 kinases; and SP600125 (5 μM), JNK kinase inhibitor. Data represent the mean value ± SEM for three separate experiments with four to six replications. ***p < 0.001 versus control SH-SY5Y cells, ^###^ *p* < 0.001, ^##^ *p* < 0.01, ^#^ *p* < 0.05 versus C2-ceramide treated SH-SY5Y cells by using one-way ANOVA followed by the Newman-Keuls post hoc test

**Fig. 4:**
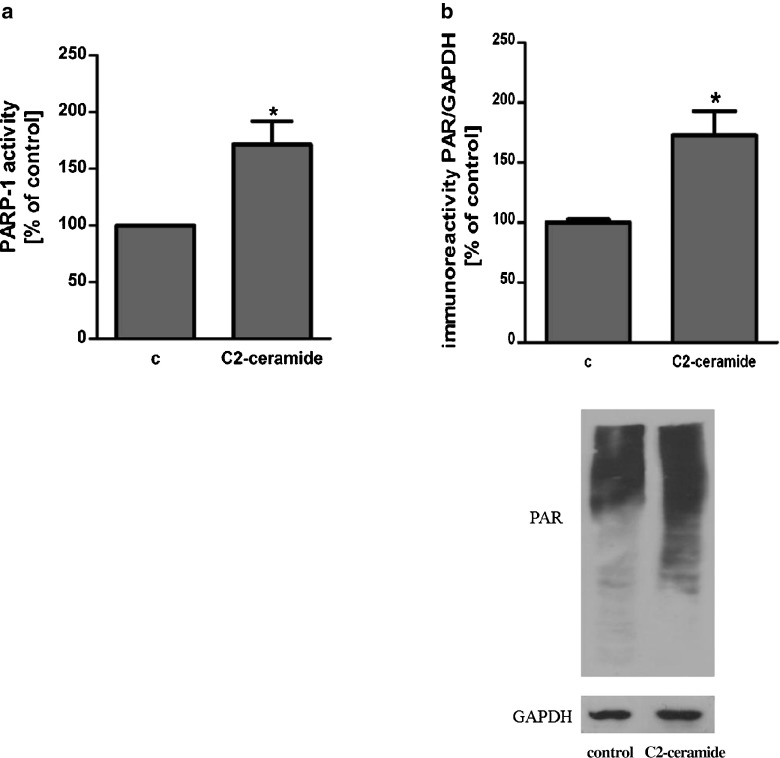

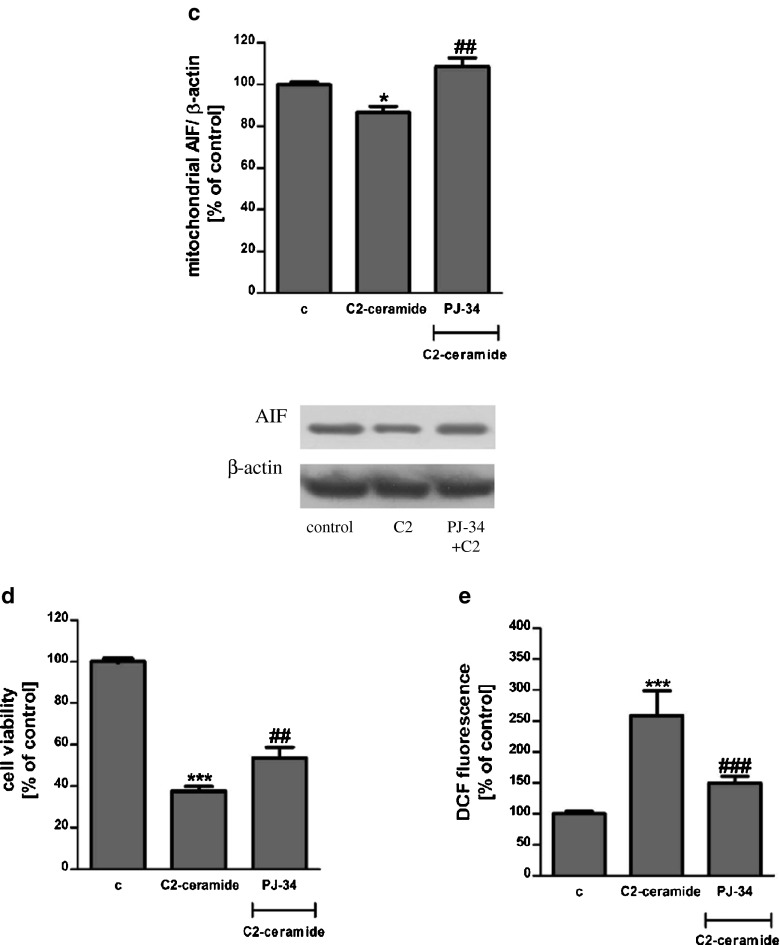
The role of PARP-1 in ceramide toxicity. PARP-1 activity and its role in oxidative stress and neuronal cell death evoked by C2-ceramide (25 μM, 24 h) (**a**). Data represent the mean value ± SEM for three separate experiments with three replications. *p < 0.0367 versus control SH-SY5Y cells by Student’s *t* test. The effect of C2-ceramide (25 μM, 12 h) on PAR immunoreactivity in SH-SY5Y cells (**b**). Data represent the mean value ± SEM for three separate experiments normalised against GAPDH. Representative Western blots from one typical experiment are shown below the graphs. *p < 0.0419 versus control SH-SY5Y cells by Student’s *t* test. The effect of PARP-1 inhibitor (PJ-34 {20 μM}) on AIF immunoreactivity in the mitochondrial fraction of SH-SY5Y cells after C2-ceramide treatment (25 μM, 12 h) (**c**). Representative Western blots from one typical experiment are shown below the graphs. Data represent the mean value ± SEM for three independent experiments normalised against β-actin. *p < 0.05 versus control SH-SY5Y cells, ^##^ *p* < 0.01 versus C2-ceramide treated SH-SY5Y cells by using one-way ANOVA followed by the Newman-Keuls post hoc test. The effect of PARP-1 inhibitor (PJ-34 {20 μM}) on SH-SY5Y cell viability (**d**) and ROS generation (**e**) after incubation with C2-ceramide (25 μM, 24 h). Data represent the mean value ± SEM for three separate experiments with four to six replications (**d**) and for three separate experiments with three replications (**e**). ***p < 0.001 versus control SH-SY5Y cells, ^###^ *p* < 0.001, ^##^ *p* < 0.01 versus C2-ceramide treated SH-SY5Y cells by using one-way ANOVA followed by the Newman-Keuls post hoc test

**Fig. 5:**
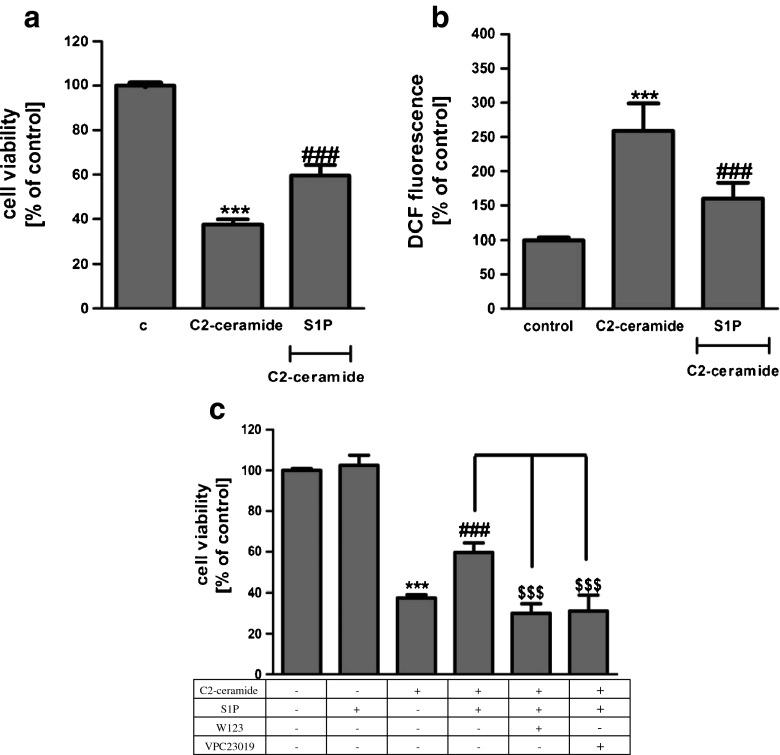
The effect of sphingosine-1-phosphate (S1P {1 μM}) on SH-SY5Y cell viability (**a**) and ROS generation (**b**) after incubation with C2-ceramide (25 μM, 24 h). Data represent the mean value ± SEM for three separate experiments with four to six replications (**a**) and for three separate experiments with three replications (**b**). The effect of S1P receptor antagonists (S1PR1-W123 {20 μM} and S1PR3–VPC23019 {1 μM}) on SH-SY5Y cell viability after C2-ceramide treatment (25 μM, 24 h) (**c**). ***p < 0.001 versus control SH-SY5Y cells, ^###^ *p* < 0.001 versus C2-ceramide treated SH-SY5Y cells, ^$$$^ *p* < 0.001 versus C2-ceramide and S1P treated SH-SY5Y cells by using one-way ANOVA followed by the Newman-Keuls post hoc test

**Fig. 6:**
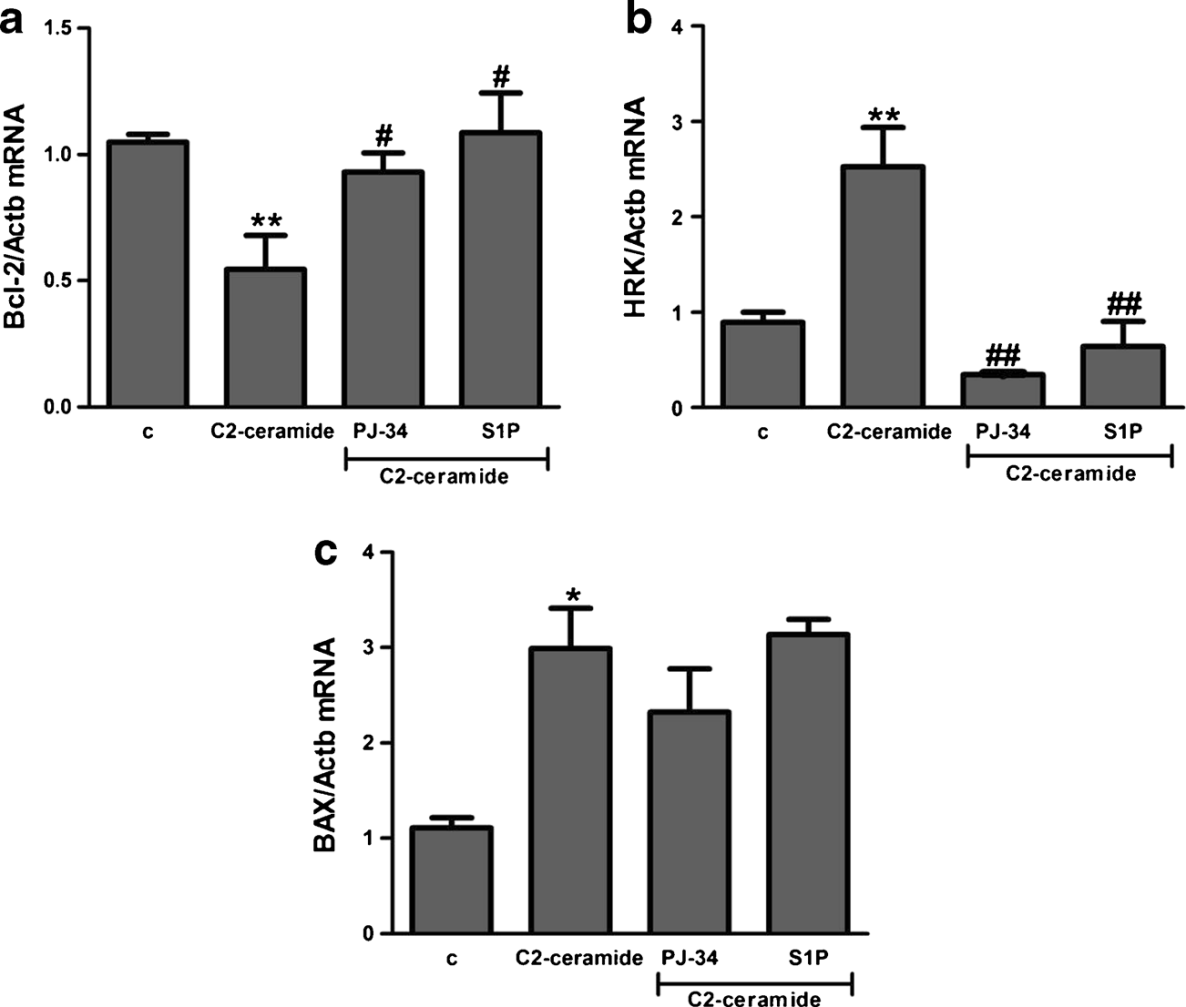
Bcl-2 protein family expression in SH-SY5Y cells subjected to oxidative stress. mRNA levels for Bcl-2 (**a**), Hrk (**b**) and Bax (**c**) were evaluated using real-time PCR after incubation with C2-ceramide (25 μM, 3 h). The value expresses the fold of gene stimulation normalised against Actb (β-actin). Data represent the mean value ± SEM for three separate experiments with three replications. **p < 0.01, *p < 0.05 versus control SH-SY5Y cells, ^##^ *p* < 0.01, ^#^ *p* < 0.05 versus C2-ceramide treated SH-SY5Y cells by using one-way ANOVA followed by the Newman-Keuls post hoc test

**Fig. 7:**
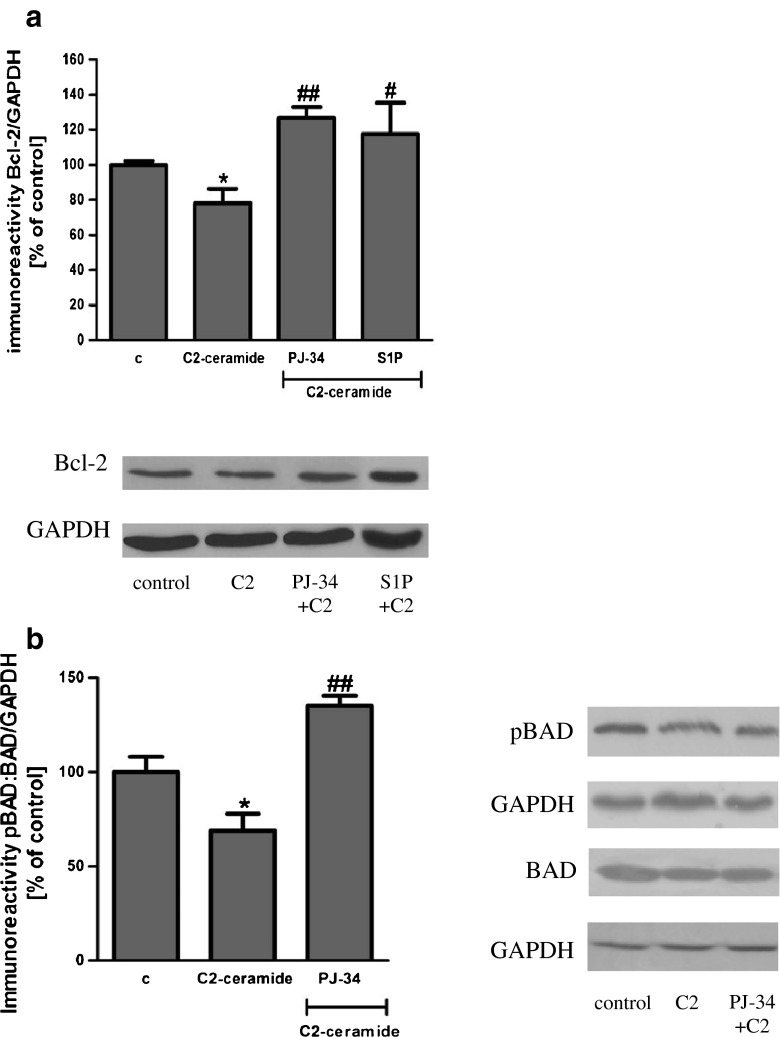
The effect of PARP-1 inhibitor (PJ-34 {20 μM}) and S1P (1 μM) on the Bcl-2 (**a**), pBad (Ser136) and Bad (**b**) protein level after incubation with C2-ceramide (25 μM, 24 h). Data represent the mean value ± SEM for three separate experiments normalised against GAPDH. Representative Western blots from one typical experiment are shown below the graphs. *p < 0.05 versus control SH-SY5Y cells, ^##^ *p* < 0.01, ^#^ *p* < 0.05 versus C2-ceramide treated SH-SY5Y cells by using one-way ANOVA followed by the Newman-Keuls post hoc test

**Fig. 8:**
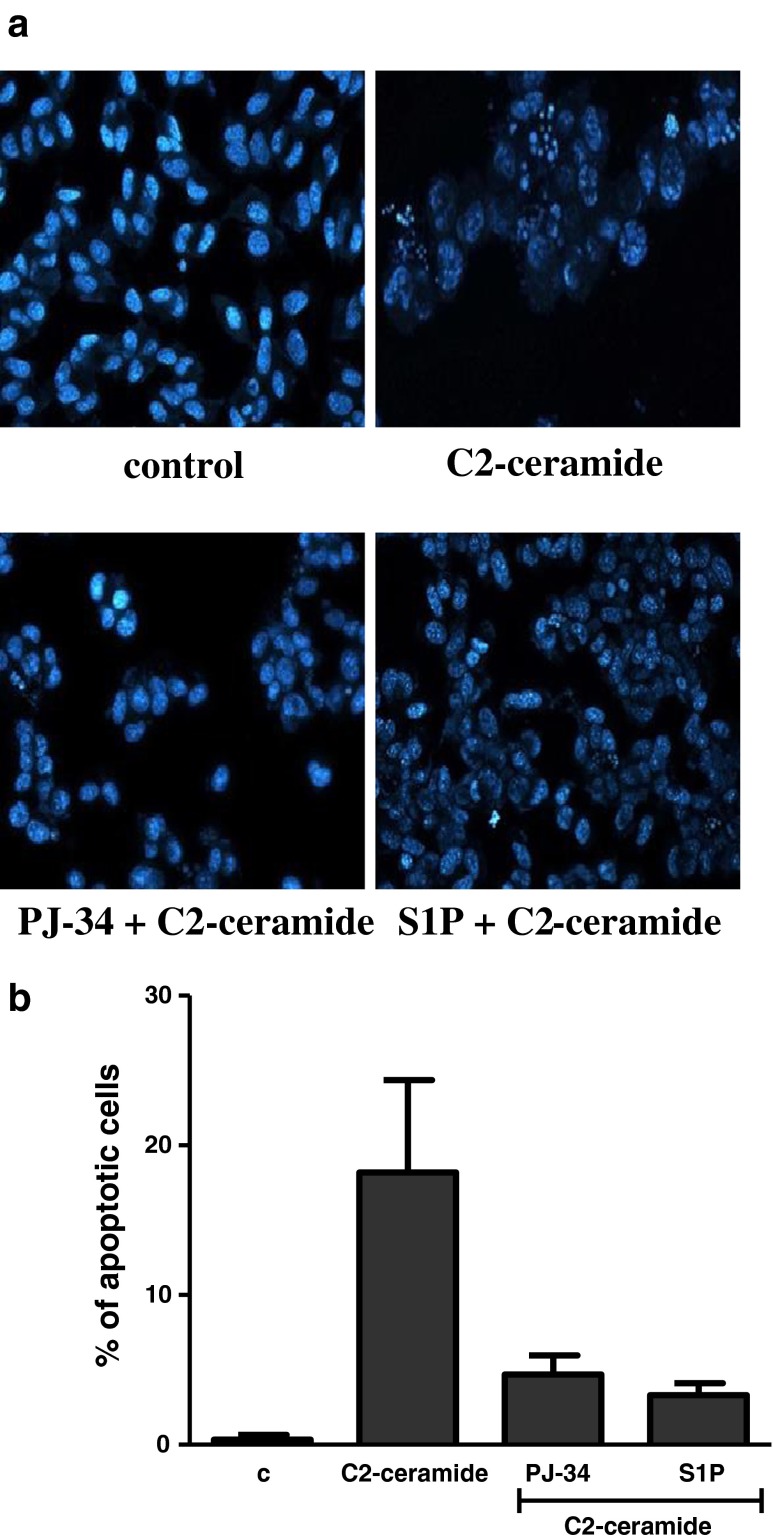
Microscopic examination of cell nuclei, stained with DNA-binding fluorochrome Hoechst 33342. The cells were treated with PARP-1 inhibitor (PJ-34 {20 μM}), sphingosine-1-phosphate (1 μM) and then with C2-ceramide (25 μM) (**a**). Cells with typical apoptotic nuclear morphology (nuclear shrinkage, chromatin condensation) were identified and counted. The results were expressed as percentages of apoptotic cells in the whole cell population from one exemplary experiment in four to eight replications (**b**)

SH-SY5Y cells were exposed for 24 h to cell-permeable C2-ceramide. After treatment, cell viability was evaluated by using MTT assay. The addition of C2-ceramide increased the level of free radicals and caused neuronal cell death in a concentration-dependent manner. For further experiments, C2-ceramide at a concentration of 25 μM used enhancing oxidative stress by 260 % of the control level and significantly decreasing cell viability by about 60 % (Fig. 1a, b). Our results indicated that the caspase-3 inhibitor (Z-DEVD-FMK) did not attenuate the ceramide-induced SH-SY5Y cell death (data not shown). Signalling through the PI3-K/Akt pathway is critical for cell survival. We observed that C2-ceramide inactivated the PI3-K/Akt pathway, thus leading to lower phosphorylation of glycogen synthase kinase-beta (GSK3β) on serine 9, its activation and cell death. Exposure to the PI3-K inhibitor (LY294002) alone or together with C2-ceramide caused SH-SY5Y cell death. However, LY294002 did not enhance cell death induced by C2-ceramide (Fig. 2a, b). Oxidative stress can lead to mitochondrial dysfunction and activation of the p53-dependent signalling pathway. Therefore, we investigated the specific inhibitor of p53 (α-pifithrin) and the mitochondrial mega-channel (cyclosporine A). Cyclosporine A and α-pifithrin enhanced neuronal cell survival after C2-ceramide treatment (Fig. 3). These compounds also significantly reduced the level of free radicals (data not shown). Numerous other cytoprotective compounds were also examined in our experimental conditions. The data show that the ERK1/2 inhibitor (U0126) and JNK inhibitor (SP600125) significantly protected the cells against death evoked by C2-ceramide (Fig. 3). Oxidative stress induced by ceramide can lead to DNA damage and to activation of the nuclear enzyme PARP-1. It was observed that C2-ceramide increased PARP-1 activity up to 170 % of the control level (Fig. 4a). In the next step, PAR immunoreactivity was evaluated. Our data demonstrated that C2-ceramide significantly increased the PAR level (Fig. 4b). Consequently, our data demonstrated that the level of AIF in the mitochondria was decreased (Fig. 4c). This effect was more pronounced in cells treated with 50 μM ceramide (data not shown). A specific PARP-1 inhibitor, PJ-34, maintained the AIF level in the mitochondria and enhanced cell viability (Fig. 4c, d). We also examined the effect of PJ-34 on ROS production after 24 h of C2-ceramide treatment. Our results also demonstrated that PARP-1 inhibition significantly reduced the level of free radicals (Fig. 4e). Looking for a novel, promising neuroprotectant, we used in the following study S1P, a product of Sphk1 enzymatic activity, which was expected to activate the pro-survival signalling pathway. Exogenously added S1P at 1 μM concentration increased SH-SY5Y cell viability and decreased the ROS level after C2-ceramide treatment (Fig. 5a, b). The question arises as to what are the possible mechanisms by which S1P enhances neuronal cell viability affected by C2-ceramide. Using a specific receptor antagonist (S1PR1-W123 and S1PR3-VPC23019), it was observed that the neuroprotective effect of S1P was in part receptor-dependent (Fig. 5c). Moreover, in the investigated model, we examined the gene expression/protein level of anti- and pro-apoptotic Bcl-2 proteins. Our data showed that the anti-apoptotic Bcl-2 mRNA level decreased, but that the pro-apoptotic Bax and Hrk mRNA level increased after C2-ceramide treatment. PARP-1 inhibition and exogenously added S1P enhanced the Bcl-2 mRNA/protein level and decreased the Hrk mRNA level affected by ceramide. However, PJ-34 and S1P had no effect on the Bax mRNA level (Fig. 6a–c). We also observed that C2-ceramide decreased the level of the Bcl-2 protein and induced dephosphorylation of the pro-apoptotic Bad protein on Ser136, which was reversed by PARP-1 inhibition (Fig. 7a, b). For evaluation of apoptotic cell death, microscopic examination of cell nuclei stained with DNA-binding fluorochrome Hoechst 33342 was used. The data showed that SH-SY5Y cells exposed to C2-ceramide presented apoptotic morphology, i.e. condensation of chromatin and nuclear fragmentation. The protective effect of PJ-34 and S1P was observed (Fig. 8). It is worth to underline that C2-ceramide significantly increased LDH release. However, we have not found any protective effect of PJ-34 and S1P (data not shown).

## Discussion

In our current study, the mechanism of ceramide-evoked neuronal death was analysed. Moreover, the effect of exogenous S1P in molecular alterations induced by ceramide in neuronal cells SH-SY5Y was evaluated. We demonstrated that C2-ceramide, through inhibition of PI3K/Akt, influenced the phosphorylation state of GSK3β and Bad. Neuronal cell death evoked by ceramide is probably connected with PI3K/Akt inhibition, which was demonstrated by us using the PI3K inhibitor. These observations are consistent with a previous study on SH-SY5Y cells [37]. It was also reported that the Akt pathway is down-regulated by C2-ceramide in the NGF-treated PC12 cells as a result of enhanced dephosphorylation of Akt1 by protein phosphatase (CAPP-PP2A) [42]. It is known that Akt phosphorylates and inactivates the pro-apoptotic Bad protein. This process can contribute to stabilisation of the mitochondrial membrane. It has been previously reported that PARP-1 inhibition induced Akt phosphorylation and activation [43]. Our study showed that PARP-1 inhibition reversed the effect of C2-ceramide on Bad phosphorylation probably due to Akt activation.

Ceramide can exert its effect by modulation of lipid kinases, e.g. JNK [44, 45]. A correlation between increased ceramide level and JNK activation via phosphorylation has been reported in differentiated PC-12 cells [46]. JNK activation and its nuclear translocation in neurons have been reported in vivo in several pathological conditions [44]. The potential target of pro-apoptotic signalling by JNK is the transcription factor c-Jun. An increase of c-Jun phosphorylation was found in neuronal nuclei after ceramide treatment [8]. Our results demonstrated that the inhibition of JNK had a small but statistically significant effect on cell viability in this stress condition. It has been reported that the JNK signalling pathway can mediate apoptosis by regulating the expression of pro-apoptotic Bcl-2 family proteins [47, 48]. Moreover, Chen et al. [49] demonstrated that amyloid beta peptides (Aβ), by activation of neutral sphingomyelinase and ceramide generation, may up-regulate death protein 5 (DP5/Hrk-harakiri) via JNK action. They observed that the both Aβ and C2-ceramide rapidly induced Hrk gene expression, increased JNK phosphorylation and AP-1 DNA binding. It is known that AP-1 DNA-binding consensus sequences are present in the promoter region of DP5/Hrk. Our finding that C2-ceramide up-regulated Hrk gene expression is in agreement with this study. Hrk was originally identified as a pro-apoptotic gene located at 12q13.1 [50, 51]. The role of Hrk in apoptosis has been described mainly in hematopoietic tissues and cultured neurons [51, 52]. It has been observed that Hrk interacts with the anti-apoptotic proteins Bcl-2 and Bcl-X_L_, but not with pro-apoptotic Bax or Bak [53] and that apoptosis by Hrk required the expression of Bax [52]. We provided evidence, for the first time, that PARP-1 inhibition reduced the mRNA level of Hrk after C2-ceramide treatment. Recent data have demonstrated that PARP inhibitors induced the suppression of JNK in vitro and in vivo [54, 55]. Therefore, it can be inferred that the effect of the PARP-1 inhibitor (PJ-34) on Hrk gene expression is associated with the attenuation of JNK activation. We demonstrated that C2-ceramide induced production of reactive oxygen species in a concentration-dependent manner. It is known that DNA damage caused by oxidative stress may activate PARP-1, a key nuclear enzyme involved in DNA repair. Our results showed PARP-1 activation and PAR polymer formation after C2-ceramide treatment. Upon activation, PARP-1 transforms nicotinamide adenine dinucleotide (NAD+) into long PAR polymers and transfers them to a variety of nuclear proteins, e.g. histones, DNA polymerases and PARP-1 itself [21, 56–59]. The basal levels of PAR are very low; however, excessive activation of PARP-1 leads to a 10- to 500-fold increase in PAR polymer formation. The PAR polymer participates directly in cell death signalling. PAR is responsible for mitochondrial AIF release and caspase-independent apoptosis [21, 57, 58, 60]. The proper AIF level in mitochondria is very important for the integration of proteins of electron transport complexes. AIF is proposed to regulate the respiratory chain indirectly, through assembly and/or stabilisation of complexes I and III [61–63]. The reduction in assembled complex I associated with AIF deficiency is anticipated to have a profound effect on mitochondrial function in neurons [64]. However, we ought to keep in mind that AIF after translocation to nucleus exerted endonuclease activity and is responsible for the DNA degradation into 50 kb fragments [65]. Our data indicated that C2-ceramide caused the AIF release from mitochondria, which was also observed in other experimental conditions by [21–23, 37, 60, 65]. Our study showed that PARP-1 inhibition significantly protected SH-SY5Y against ceramide-induced cell death by reducing the level of ROS production, thus preventing the AIF release from the mitochondria and increasing the mRNA level of anti-apoptotic Bcl-2. Moreover, we observed enhancement of Bad phosphorylation and down-regulation of Hrk gene expression. A previous study by Kauppinen et al. [66] showed that pharmacological inhibition of ERK1/2 prevented PARP-1 activation and reduced PARP-1-mediated neuronal death. Our results demonstrated that the inhibition of ERK1/2 kinases had a small but statistically significant effect on cell viability. Because it is postulated that ceramide and S1P play a crucial role in cell survival and death, the effect of exogenous S1P was evaluated. It has been observed that S1P (1 μM) exerts its inhibitory effect on apoptosis through members of the Bcl-2 protein family and the reduction of oxidative stress. The present findings implicate a decrease in the mRNA level of the pro-apoptotic (Hrk) proteins and an increase in the mRNA/protein level of anti-apoptotic (Bcl-2) Bcl-2 proteins as one of the mechanisms through which S1P protects SH-SY5Y cells from apoptosis. These data are consistent with studies on other cell types. It has been observed that exogenous S1P regulates the expression of pro-apoptotic and anti-apoptotic proteins; e.g. exogenous S1P increases the expression of the anti-apoptotic Bcl-2 [67] and Mcl-1 [68] while it down-regulates the pro-apoptotic proteins Bad and Bax [69]. Exogenous S1P also blocks the translocation of Bax to the mitochondria [70]. Our study with specific S1P receptor antagonists revealed that the pro-survival signal of S1P was mainly dependent on S1PR1 and S1PR3 receptors. S1P signalling through these receptors may induce the activation of the PI3K/Akt pathway [12, 13, 71]. It has been observed that the activation of Akt can inhibit apoptosis by blocking the release of AIF from the mitochondria [37]. Our data indicate that such events as: activation of PARP-1, accumulation of PAR and alteration of AIF level in mitochondria play a significant role in the mechanism of apoptotic caspase-3 independent cells death. As a conclusive remark, we suggest that the modulators of sphingosine-1-phosphate receptor(s) and PARP-1 inhibitor(s) should be considered in the therapy of neurodegenerative disorders.
